# Patients with newly diagnosed cervical cancer should be screened for anal human papilloma virus and anal dysplasia: Results of a pilot study using a STELLA computer simulation and economic model^☆^

**DOI:** 10.1016/j.pvr.2017.12.001

**Published:** 2017-12-13

**Authors:** Eli D. Ehrenpreis, Dylan G. Smith

**Affiliations:** a University of Chicago, President E2Bio Consultants, 2906 Central St, Evanston, IL 60201, USA; b TekLink International, Warrenville, IL, USA

## Abstract

**Background:**

Women with cervical cancer often have anal human papillomavirus (HPV) infection and anal dysplasia. However, effectiveness of anal HPV screening is unknown.

**Methods:**

A dynamic model was constructed using STELLA. Populations are represented as "stocks" that change according to model rates. Initial anal cytology in new cervical cancer patients, dysplasia progression and regression, cost of treating high-grade squamous intraepithelial lesions (HSIL), and lifetime costs for anal cancer care were extrapolated from the literature. Local costs of anal HPV testing and cytology were obtained. Outcomes included anal cancer rates, anal cancer deaths, screening costs and cancer care.

**Results:**

Benefits in the screened group included reduction in anal cancers after three years and anal cancer deaths after four years. After 10 years, predicted costs per anal cancer prevented and anal cancer deaths were $168,796 and $210,057 and were $98,631 and $210,057 at 20 years. Predicted costs per quality of life year saved at 10 and 20 years were $9785 and $1687. Sensitivity analysis demonstrated cost-effectiveness of screening for a variety of cure rates HSIL with electrocautery.

**Conclusion:**

Screening for anal HPV and treatment of anal HSIL in patients with cervical cancer is cost-effective, prevents anal cancer and reduces anal cancer deaths.

## 1 Introduction

Chronic anal infection with human papillomavirus (HPV) is a known risk factor for anal carcinoma. The pathophysiology of HPV-associated carcinoma involves sexually-transmitted infections with high-risk HPV subtypes, chronic infection, altered immunity and a dysplasia to carcinoma sequence [2], [3]. Because cost-effective methodologies exist to identify anal HPV infection, screening strategies can prevent the development of anal cancer [3]. At present, routine screening for anal dysplasia and cancer is limited to HIV-infected patients and men having sex with men (MSM) [4], [5]. The economics and clinical benefits of anal HPV screening in women with cervical HPV infection is not clearly defined [6]. Only one national society guideline has recommended anal cancer screening in women with abnormal cervical cytology [7], even though women with cervical HPV are seven times more likely to have an anal HPV infection [8]. Furthermore, almost 50% of women with cervical high-grade squamous intraepithelial lesion (HSIL) or microinvasive cancer have anal HPV infections [9]. Because the vast majority of cervical and anal cancers result from the same high-risk HPV subtypes, identifying patients with these infections is a high priority. In this study, a dynamic model was developed to estimate anal histologic outcomes in women with histories of cervical cancer and to examine the effects of screening for anal HPV on the costs and development of anal cancer.

## 2 Methods

### 2.1 Model development

**Fig. 1:**
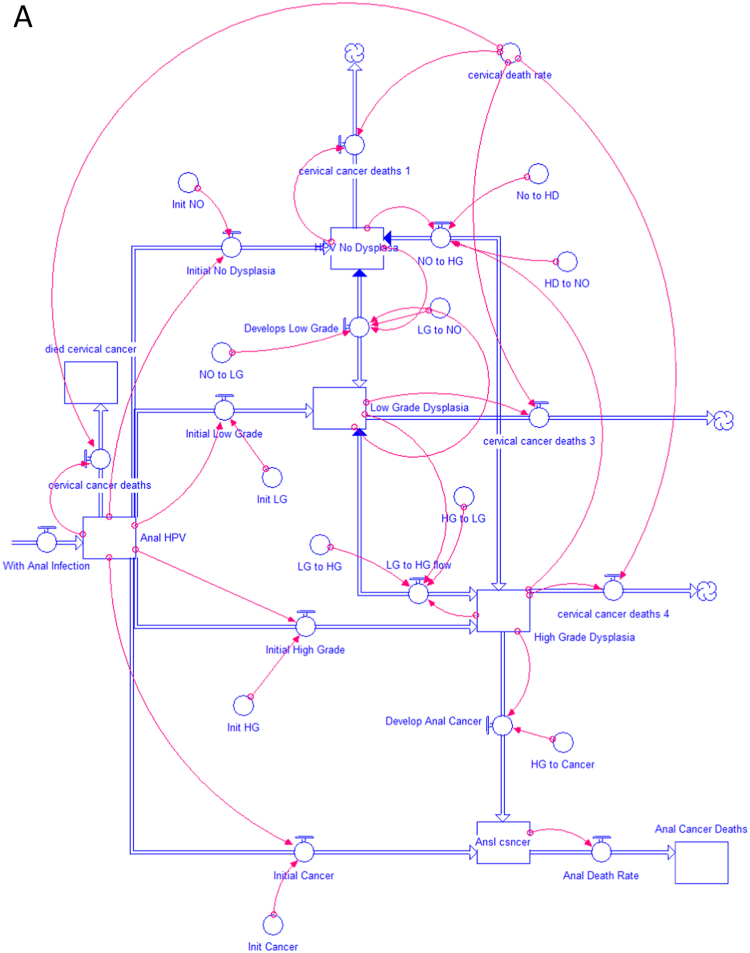

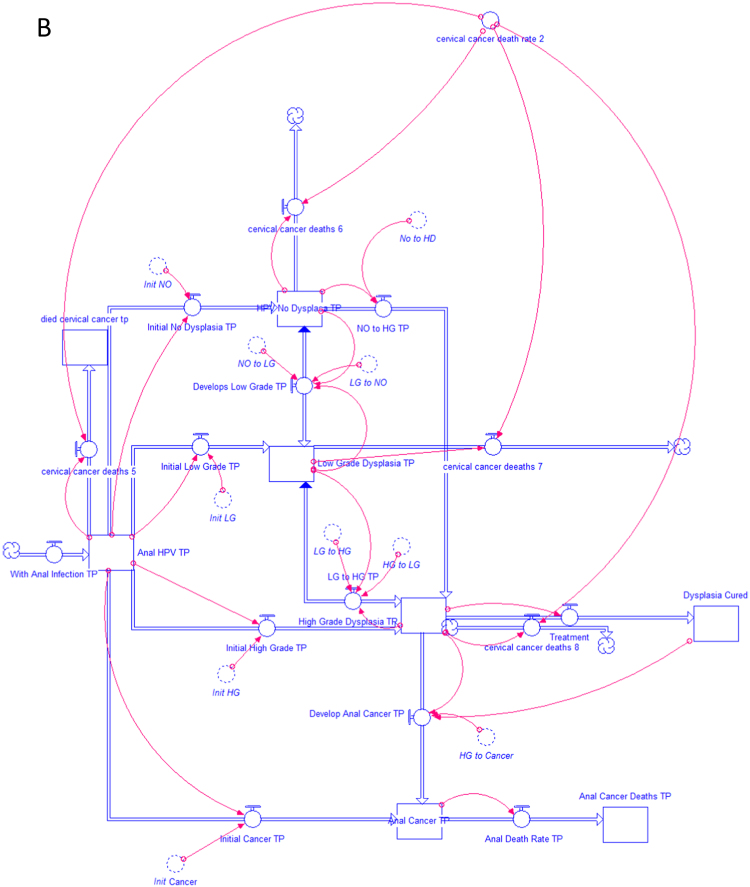
(A) Population of women with cervical cancer with no screening for anal HPV. (B) Population of women with cervical cancer screened for anal HPV.

A dynamic model was constructed to simulate the population of patients with cervical cancer and the expected changes occurring in anal histology over time using STELLA software (Systems Thinking, Experimental Learning Laboratory with Animation, isee systems, Inc., Lebanon NH, USA). STELLA models are graphically-based, continuous simulations of complex processes. The program has been utilized by a variety of disciplines to analyze dynamic processes consisting of continuous flows of materials, resources or individuals that vary over time [10]. Models are characterized by state variables and control variables. In the current model, state variables represent the number of patients in a particular group, for example, those with HSIL (termed high-grade dysplasia-HGD in the model format). The cumulative numbers of patients in each stage of anal disease are represented as reservoirs or ‘stocks’ in the STELLA model. Control variables represent rates of change within the model and update the value of state variables during each time period. In STELLA terminology, the interaction between reservoirs is regulated by these control variables or ‘flows’ that are graphically connected to interacting reservoirs. The rates for these ‘flows’ are represented as “converters” that attach to appropriate stocks and flows using ‘connectors’ to modify flow rates. The use of these converters allows for easy modification of flow rates throughout the entire model. In this model, flows represent the progression of patients with normal anal histology (no dysplasia) to low grade dysplasia (LGD), HSIL and anal cancer. Each of these populations is graphically represented by reservoirs. The mathematical model that underlies the simulation is constructed as a set of differential equations. The study model is represented in Model Fig. 1A and B. The first model, shown Figure A, represents the unscreened population of patients newly diagnosed with cervical cancer and their progression to anal HPV infection, dysplasia, cancer and anal cancer death [11]. This is the current clinical approach in patients with cervical cancer and has not undergone rigorous cost analysis. The second model, shown in Figure B, also simulates all new patients with cervical cancer. In this group, all undergo an initial screen for high risk anal HPV infection and tissues is collected for cytology. The model assumes that 48.3% of these patients will be infected with high-risk anal HPV subtypes [9]. These 5555 patients with high-risk anal HPV infection, undergo anal cytologic analysis (anal cytology), and become the group of patients continuously evaluated in the system. A simulation was performed for the approaches demonstrated in the two models that continued over a 20-year period.

The results of the initial anal cytology studies are based on recent work by Cronin et al. [1]. The predicted progression from anal HPV to low grade dysplasia (LGD) to high-grade squamous intraepithelial lesion (HSIL) and anal cancer (and reversion from these stages) were acquired from the literature [1], [12]. Information on progression to the various forms of dysplasia not available in HPV-infected women was obtained from literature on HIV non-infected MSM [13], [14], [15]. Patients with abnormal cells of undetermined significance (ACUS) were divided into the aforementioned groups per data from Goldie, et al. as follows: normal=58%, LGD=24%, HSIL=18% [4]. Simulations were performed with annual input of 5555 new patients with 12 month iterations. The model assumes no regression back to the HPV negative (not tested) population, so all HPV positive women undergo annual anal cytologic examinations. Ongoing deaths from cervical cancer diminishes patient stocks with each iteration of the model, assuming cervical cancer as a primary cause of death ends after 10 years. Results of the initial anal cytologic examination were used to determine the number of patients in each stock [1], representing the initial conditions of the system. Based on current literature, we anticipated a 95% anal cancer prevention rate with high resolution anoscopy and electrocautery techniques in women with anal HSIL [16]. All model assumptions are shown in Table 1. Equations incorporated in the model are shown in Supplementary Table A. Results based on initial simulations are reported in this study.

**Table 1:** Model assumptions: screening for Anal HPV and anal dysplasia in patients with cervical cancer.

Annual Incidence of cervical cancer in the US attributable to HPV = 11,500 (reference [11])
% of woman with history of cervical high grade dysplasia or microinvasive cancer with anal HPV=48.3% (reference [9])
Total number of woman with woman with history of cervical high grade dysplasia or microinvasive cancer with anal HPV=5555
Average age at time of diagnosis of cervical cancer=49 (ref [37])
% of women with history of genital cancer initially having anal ASC-US=19.4% (reference [1])
% of women with history of cervical cancer initially having no anal dysplasa=50.8% (reference [1])
% of women with history of high grade cervical cytology initially having anal low grade dysplasia=16.0 (reference [1])
% of women with history of genital cancer initially having anal high grade dysplasia=3.0% (reference [1])
% of women with history of high grade cervical cytology initially having anal low Grade dysplasia=2.3% (reference [1])
% of women with anal HPV and no dysplasia that develop high grade dysplasia over two years=8% (Based on MSM) (reference [12])
% of women with anal HPV and low grade dysplasia that develop high grade dysplasia over two years=36% (based on MSM) (reference [12])
% of women with anal HPV and ASC-US that develop high grade dysplasia over two years=62% (reference [13])
ACUS cytology equivalents (Based on 1 year follow up cytology in MSM) (reference [4])
ACUS represents normal= 58%, ACUS represents LGD=24%, ACUS represents HSIL= 18%, ACUs represents cancer=0
Histologic progression and regression (Based on 1 year follow up in MSM), (references [4], [15])
Progression: Normal to LGD=1.9%, Normal to HSIL=1.78%, Normal to cancer=0%, LGD to HGD=16.5%, LGD to anal cancer=0.05% (estimated), HSIL to anal cancer=3.6%
Regression: LGD to normal=22.65%, HSIL to LGD=22% (estimated), HSIL to normal=11.36%
Annual anal cancer death rate = 6.72% (reference [17])
Five year anal cancer death rate=33.6% (reference [17])
Annual death rate from cervical cancer=6.4% (reference [4])
Five-year death rate from cervical cancer=32% (reference [4])
Average life expectancy for a female age 49=34 years (reference [38])
Quality of life weight adjustment for anal cancer=0.56 (reference [4])
Quality of life weight adjustment after treatment for anal HSIL=0.9
Quality of life weight adjustment for cervical cancer = 0.70 (0.79–0.62 for Stage 1 and Stage 2) (ref [39])
Quality adjusted life expectancy woman age 49=21.6 (38)
Quality adjusted life expectancy woman age 49 surviving cervical cancer=21.6 × 0.7=15.12
Quality adjusted life expectancy woman age 49 surviving cervical cancer treated for anal HSIL=15.1 × 0.9 = 13.6
Costs of Individual Tests and Treatments Used for Model Calculations
HPV screen=$50.27
Anal cytology =$37.94
Treatment of anal HGD=$3597.00
High resolution anoscopy and biopsy=$147.68
Cost of cancer treatment=$60,913.00 (reference [15])
Costs are annualized with estimated 2.05% inflation rate in the model

#### 2.1.1 Cost analysis

Costs for patients receiving no screening and those receiving screening and treatment included the cost of care for patients with anal cancer. Costs for anal high-risk HPV testing ($50.27) and anal cytology ($37.94) were obtained from the NorthShore University HealthSystem. All costs were adjusted for an annual inflation rate of 2.05%, (see Table 3).

#### 2.1.2 Sensitivity analysis

Because the overall benefit of the proposed screening program are highly dependent on the effectiveness of curative treatment for anal high-grade dysplasia, sensitivity analysis was performed for a range of potential cure rates for electrocautery treatment for treatment of anal high-grade dysplasia. Outcomes were determined for cure rates of 38%, 48%, 58%, 78%, 88% and 98%.

### 2.2 Outcomes determined from the model

Overall outcomes, included numbers of subjects with new and cumulative anal cancers, new and cumulative anal cancers and costs per anal cancer, anal cancer deaths prevented and cost for quality adjusted life years. These factors determined by the simulation performed provide the rational for screening this patient population.

## 3 Results

**Fig. 2:**
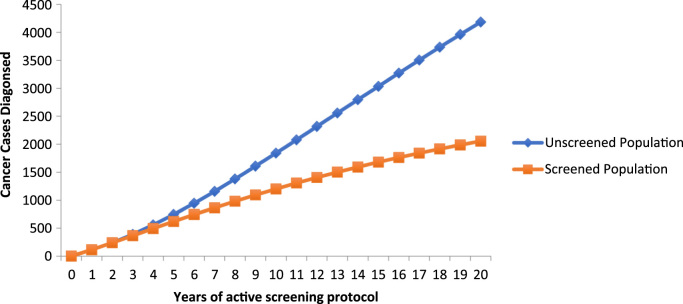
Model prediction for cumulative cases of anal cancer in the screened vs. unscreened population of women with a history of cervical cancer.

**Fig. 3:**
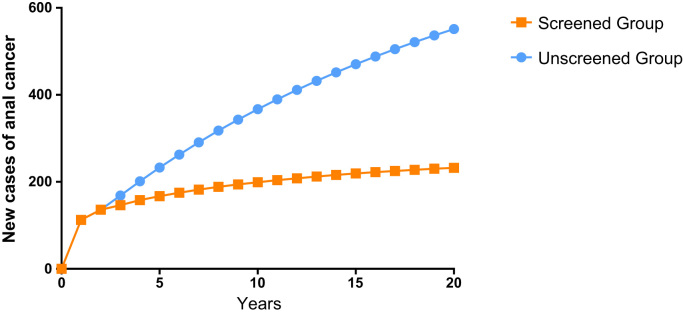
Predicted number of new anal cancer cases in patients with cervical cancer undergoing screening for anal dysplasia or no screening.

**Fig. 4:**
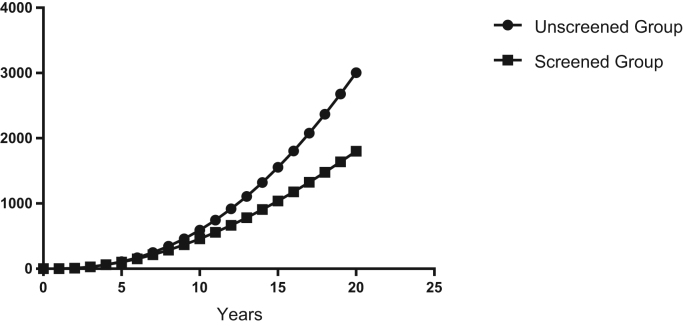
Model prediction for cumulative cases of anal cancer in the screened vs. unscreened population of women with a history of cervical cancer.

The model shows that screening lowers cumulative numbers of diagnosed cancer cases after three years (388 cases in Group 1 vs 366 cases in Group 2). Differences in cumulative anal cancer cases between the groups increase after that time. After 10 years, the model predicts 1841 cumulative anal cancers in Group 1 and 1202 anal cancers in Group 2 and 4184 cumulative anal cancers in the Group 1 and 2055 anal cancers in Group 2 after 20 years (Table 2, Fig. 2). As seen in Fig. 2, after year five, the rate of development of anal cancer in Group 2 begins to decline while cases in Group 1 rise exponentially. This occurs as the number of new cases of anal cancer decreases in Group 2 (Fig. 3). Cancer deaths are reduced in Group 2 after 4 years, followed by accentuated differences between unscreened and screened groups (Table 2, Fig. 4).

**Table 2:** Model estimates of cumulative anal cancers and anal cancer deaths in cervical cancer patients that are screened and treated or not screened and treated for anal HPV and dysplasia.

Year	Cumulative anal cancers Unscreened population	Cumulative anal cancers	Cumulative anal cancer deaths	Cumulative anal cancer deaths
		Screened population	Unscreened population	Screened population
0	0	0	0	0
1	113	113	0	0
2	239	239	9	9
3	388	366	29	29
4	557	494	61	60
5	744	620	108	101
6	945	743	169	152
7	1157	864	248	214
8	1379	980	344	285
9	1607	1093	458	367
10	1841	1202	592	458
11	2078	1306	744	557
12	2317	1406	917	666
13	2557	1501	1109	782
14	2796	1593	1321	907
15	3034	1680	1554	1039
16	3271	1762	1805	1179
17	3504	1841	2077	1325
18	3735	1916	2368	1478
19	3961	1987	2678	1637
20	4184	2055	3006	1802

Cost differences between the two groups are shown in Table 3. After five years, the estimated costs in Group 1 and Group 2 were $54,418,071 and $73,650,033 respectively, (increased Group 2 cost of $19,231,962). At 10 years, the increased cost in Group 2 was $28,357,665 and was $31,463,030 after 20 years. These cost differences translated to $3125,027, $210,057 and $26,133 per anal cancer prevented after 5,10 and 20 years respectively. Costs per anal cancer death prevented at 5 years was $1107,194 and was $151,475 and $26,113 at 10 and 20 years respectively. The costs per life year saved in the screened group was $64,372 at 5 years, $8807 at 10 years and $1518 at 20 years. Cost per quality of life year saved after 5 years in the Group 2 was $71,229 and was $9785 and $1687 after 10 and 20 years respectively. Sensitivity analysis demonstrated the effectiveness and cost effectiveness of screening throughout the examined range of cure rates of HSIL with electrocautery (Table 4, Table 5).

**Table 3:** Estimates of costs from dynamic modeling for screening for HPV and treatment of anal dysplasia in patients with cervical cancer.

	Costs in unscreened group (Cancer care only)	Costs in screened group (HPV screening, annual cytology, treatment of HSIL, and cancer care)	Cost Difference	Cost per anal cancer prevented	Cost per anal cancer death prevented	Cost per quality of life year saved
5 years	$54,418,071	$73,650,033	$19,231,962	$291,409	$3205,127	$71,229
10 years	$165,859,480	$194,217,145	$28,357,665	$168,796	$210,057	$9785
20 years	$536,526,508	$567,969,558	$31,463,030	$98,631	$26,133	$1687

**Table 4:** Results of sensitivity analysis comparing costs per life year saved for six different cure rates of anal high-grade squamous intraepithelial lesions (HSIL) with electrocautery techniques.

Cure rate of anal high- grade dysplasia	Cost/life year saved	Cost/life year saved	Cost/life year saved
	5 years	10 years	20 years
0.38	$321,194.20	$66,578.65	$27,398.40
0.48	$209,408.02	$41,747.47	$16,634.34
0.58	$150,733.67	$28,669.60	$10,862.67
0.78	$91,251.41	$15,320.73	$4869.95
0.88	$74,457.50	$11,504.29	$3128.97
0.98	$64,371.75	$8806.70	$1518.24

**Table 5:** Sensitivity analysis comparing the percent reduction in new anal cancers and anal cancer deaths for six different cure rates of high-grade squamous intraepithelial lesions (HSIL) with electrocautery techniques.

Cure rate of anal high- grade dysplasia	Reduction in new anal cancers after	Reduction in anal cancer deaths after	Reduction in new anal cancers after	Reduction in anal cancer deaths after	Reduction in new anal cancers after	Reduction in anal cancer deaths after
	5 years	5 years	10 years	10 years	20 years	20 years
0.38	12%	3%	24%	10%	35%	21%
0.48	15%	4%	30%	13%	42%	26%
0.58	19%	4%	35%	16%	47%	30%
0.78	25%	6%	42%	20%	54%	36%
0.88	27%	6%	44%	22%	57%	39%
0.98	28%	6%	46%	23%	58%	40%

## 4 Discussion

The incidence of anal HPV is increasing, and women over 50 years of age are the most commonly affected group [17]. Approximately 90% of anal cancers are a result of anal infection with HPV [2], [18]. Since anal HPV is identified with a simple and inexpensive technique, screening in HIV-infected patients and MSM is generally recognized as a cost-effective method for prevention of anal cancer [3], [4], [5]. However, there are limited clinical data proving efficacy of screening for anal HPV in any patient group [19]. At present, the testing for HPV DNA is performed from cervical cells and is not an FDA approved indication for screening for anal infection. Furthermore, even the role of digital rectal exam as a potential method to identify anal lesions in patients with cervical cancer has not been fully investigated.

Unfortunately, women that not infected with HIV have been overlooked as a group with the potential to benefit from anal HPV screening, despite the known risk of anal HPV infection and cytologic abnormalities woman with a history of cervical HPV. It is possible that anal cancer screening in this patient group is not recommended because epidemiologic evidence suggests that transient HPV infection is common in woman [8]. In addition, because anal cancer is relatively rare, the benefit and cost effectiveness of screening HPV infected women is undefined. By focusing on woman with a new diagnosis of cervical neoplasia in the current study, we reduced the scale and costs of screening the entire group.

More than 48% of women with high grade cervical epithelial lesions have anal HPV infections [5]. Infections in these patients generally come from high-risk HPV subtypes [20]. Older woman with anal HPV infections are also at higher risk for persistent anal infections [8]. Ongoing screening for anal HPV infection, and treatment of high grade anal dysplasia in patients with a history of cervical cancer has been suggested by some investigators [21], [22] but only been recommended by one medical society [3]. The utility of this approach would require a prospective clinical trial. At present, a prospective study termed the Anal Cancer HSIL Outcomes Research (ANCHOR) study is recruiting HIV-positive patients with High Grade Squamous Intraepithelial Lesion (HSIL). These patients will undergo random assignment to receive treatment for HSIL or ongoing monitoring over a five years period. The purpose of the ANCHOR study is to determine whether screening and treatment of HSIL is effective in preventing the development of anal cancer [23]. Since there is no similar study in women diagnosed with cervical cancer, another high-risk group for anal cancer, modeling of the effects of a screening program can function as an initial means of estimating both the efficacy and cost/benefits of such a program. This approach has been utilized throughout the medical literature and several cost models have previously demonstrated potential benefit of screening for anal HPV in other patient groups [4], [15]. Furthermore, it has been suggested that the application of mechanistic mathematical models that combine the biologic behavior and population dynamics of HPV infection has potential in the study of HPV-related diseases [24].

Dynamic computer simulation and modeling with STELLA software has been practiced in a number of fields including economics, psychology and environmental science [10], [25], [26]. Dynamic modeling has been applied to some studies in the medical field [27]. Cost modeling with STELLA has been utilized to evaluate outcomes and costs for care in the emergency department and for cardiovascular surgery [28], [29]. STELLA models are advantageous for investigating complex systems that may not be amenable to easy acquisition of data. Examples of the use of STELLA modeling in the medical literature include benefit estimates of treatment options in pediatric patients with Crohn's disease [30], and utility of information technology for prevention of adverse drug events [31]. Our group has previously applied STELLA modeling to predict colonic levels of topically active gastrointestinal drugs that are unmeasurable using conventional techniques [32]. We chose this method in the current study because objective values for several parameters built into the model were either not available in the medical literature, or needed to be estimated from findings in other patient groups, (such as MSM). The use of “converters” to represent rates in STELLA models facilitate the performance of sensitivity analyses over a wide range of values for these rates. This easy modification of model parameters allows for flexibility in the handling complex interrelationships throughout the system [26], [27]. Adjustment of multiple rates can be easily made throughout the model and simulations can be created to determine downstream effects of these changes (such the number of patients developing and dying from anal cancer).

Testing women with a history of cervical cancer for anal HPV and dysplasia clearly meets established appropriateness criteria for a potentially viable screening program [33]. Patients with a history of cervical neoplasia have been recently proven to represent a population at risk for persistent infection with oncogenic subtypes of HPV [34]. Similar to cervical cytologic testing [35], simple anal sampling techniques can be used to simultaneously determine the presence of high risk HPV subtypes and to evaluate for evidence of dysplasia [36]. Testing can be performed easily in the office setting, and has been validated in other high-risk groups for anal cancer. Furthermore, intervention for individuals found to have HSIL is highly effective in preventing progression to anal cancer [2], [3].

Implementation of a new screening program of this type ultimately requires high-quality clinical trials to prove effectiveness. As an initial approach, our study clearly demonstrates the benefit of screening and treatment in patients with a new diagnosis of cervical cancer. For example, after year five, the number of new cases of anal cancer in the screened group begins to decline, and with increasing differences in cancer rates between the unscreened and screened group over time. Our model predicts that after 10 years, 1841 cases of anal cancer will occur in the unscreened group and 1202 anal cancers will occur in the screened group and 4084 cases in the unscreened group and 2055 cases of anal cancer after 20 years (Table 2, Fig. 2). Reduction in death from anal cancer is predicted in the screened group as well. After 10 years, the model predicts 592 cases of anal cancer death in the unscreened group and 458 cases of anal cancer deaths in the screened group and 3006 cases of anal cancer death in the unscreened group and 1802 cases of anal cancer death in the screened group after 20 years (Table 2). These rates are based on an assumed 98% cure rate of anal high-grade dysplasia with currently available electrocautery techniques. Sensitivity analysis demonstrates that beneficial effects in this population occur even with a cure rate as low as 38% (Table 5).

Costs in the screened group include initial testing for anal HPV, anal cytologic examinations performed annually, the cost of treatment for anal HSIL and annual follow up for patients treated with HSIL (including high resolution anoscopy and biopsy). These costs are offset by the cost of cancer care for the larger number of cases of anal cancer in the unscreened group compared to the screened group. After 10 years, the model predicts that the cost for each case of anal cancer prevention was $168,796, and the cost of prevention of each death from anal cancer was $210,057. After 20 years, the predicted costs for prevention of each case of anal cancer was $98,631 and the cost of prevention for each death from anal cancer was $26,133, (Table 3). The cost of the program per quality adjusted life year at 10 years and 20 years was $9785 and $1687. These predictions represent a very efficient program for screening a patient group at high risk for anal cancer. By comparison, interventions are considered to be efficient (and therefore acceptable) if the cost of their implementation less than $50,000 to $60,000 per quality of life year gained [37], [38], [39]. Sensitivity analysis shows continuing benefits and cost benefits of screening patients with cervical cancer for anal HPV, even for cure rates of anal HSIL as low as 38% (Table 4, Table 5).

In contrast to our study, a recent cost model based on data from British Columbia, questioned the overall benefit of screening woman with cervical dysplasia for anal lesions [6]. The improved costs and benefits predicted by our model may have occurred by the addition of an initial screening for high-risk anal HPV subtypes, with follow up anal cytology only in those infected with HPV. In addition, our analysis restricted the group receiving ongoing anal cytology and treatment to only include patients with a new diagnosis of cervical cancer.

There are several limitations that need to be considered in reviewing the data presented in this study. This study is considered an initial analysis and is based on the best available data in the medical literature. Data regarding the annual occurrence of cervical cancer, deaths from cervical cancer and the findings on anal cytology in patients with a history of cervical cancer are based on recent high-quality studies. The evolution of anal cytologic changes in women with anal HPV infection has not been clearly defined, requiring data from MSM to provide some assumptions regarding these processes in the model. One conceit of the model is the simplified assumption that chronic anal infection with high-risk HPV results in a progression from LGD to HSIL to anal cancer [40]. Although studied to a greater extent in patients with cervical HPV infection, the greatest risk for the development of HPV-associated dysplasia is the presence of persistant HPV infection. Extended activity of viral oncoproteins E6 and E7 produce DNA mutations leading to anal dysplasia and anal cancer [2]. Prolonged infection may perhaps produce a variety of forms of dysplasia and cancer that arise without the sequence described in the model [41], [42]. An addition, because the spontaneous regression rate for anal HSIL to no anal HPV has only been investigated on MSM and is less than 2% [43], the model assumes that no spontaneous regression of persistant anal HPV infection will occur in woman with anal HSIL. In addition, assumptions regarding initial findings are based on a recent groundbreaking study by Cronin, et al. [1]. In that study, subjects with malignancy included women with vaginal and vulvar cancer as well as cervical cancer. The initial findings on anal cytology for all gynecologic cancers were included together, and these data were previously unavailable. For this reason, for the purposes of producing this model, an assumption was made that initial anal cytologic findings are consistent for all forms of HPV-associated cancers of the female genital tract. Finally, because the definition and natural history of ASC-US is less clearly defined in the literature, parameters in the model for ASC-US are adjusted to represent normal cytology, LGD and HSIL based on the studies evaluating one-year follow-up of ASC-US [4].

Sensitivity analyses with different rates of cure for anal HSIL adds to the quality of information obtained. Use of a dynamic model, particularly based on STELLA software also allows for sensitivity analysis of all parameters in the model. Further studies and additional simulations are planned to further clarify changes in rates within the model on predicted downstream effects, such the prevention of anal cancer. Cost estimates based on results of the model could vary considerably depending on costs within individual healthcare delivery systems. New estimations based on different costs for HPV screening, anal cytologic analysis, high resolution anoscopy, treatment for anal HSIL and cost of cancer care can easily be applied to evaluate the range of costs that incurred with the proposed screening system. Despite these considerations, the model shows that a program of screening for HPV, evaluation of anal cytology, ongoing screening and treatment for patients found to have HSIL is highly cost-efficient, is expected to save lives and prevent cases of anal cancer in women with a history of cervical cancer.

In summary, woman with a new diagnosis of cervical cancer that are screened for anal HPV infection, monitored for anal cytology, and treated for anal HSIL will benefit from prevention of anal cancer and anal cancer deaths and will have decreased cost of care. Clinical trials are needed to validate these findings. Healthcare providers are encouraged to be diligent in evaluating patients with cervical cancer having anorectal complaints. It is critical to appreciate their risk for anal HPV infection and to prevent the potential consequences of these infections.

## Author contributions

Eli D Ehrenpreis- Conception and design of the study, acquisition of data, analysis and interpretation of data, drafting and revision of the manuscript, and final approval of the version to be published.

Dylan G Smith- Study design, acquisition of data, analysis and interpretation of data, critical revision of the manuscript for important intellectual content, and final approval of the version to be published.

Dr. Ehrenpreis and Mr. Smith agree to be accountable for all aspects of this manuscript.

The authors declare that they have are no conflicts of interest related to this study.

## Conflict of interest statement

None.
