# Supplementary material for: Patients with newly diagnosed cervical cancer should be screened for anal human papilloma virus and anal dysplasia: Results of a pilot study using a STELLA computer simulation and economic model
Source: Papillomavirus Res. 2017 Dec 13;5:38–45. doi: 10.1016/j.pvr.2017.12.001 (PMC5886984; doi:10.1016/j.pvr.2017.12.001)
Supplement: Supplementary file 1 — Supplementary material [file mmc1.docx]

**Supplementary Table A**

**Model Equations: Screening and Treatment for Anal HPV and Anal Dysplasia**

Anal_Cancer_Deaths(t) = Anal_Cancer_Deaths(t - dt) + (Anal_Death_Rate) * dt

INIT Anal_Cancer_Deaths = 0

INFLOWS:

Anal_Death_Rate = 0.083*Anal_cancer

Anal_Cancer_Deaths_TP(t) = Anal_Cancer_Deaths_TP(t - dt) + (Anal_Death_Rate_TP) * dt

INIT Anal_Cancer_Deaths_TP = 0

INFLOWS:

Anal_Death_Rate_TP = 0.083*Anal_Cancer_TP

Anal_Cancer_TP(t) = Anal_Cancer_TP(t - dt) + (Develop_Anal_Cancer_TP + Initial_Cancer_TP - Anal_Death_Rate_TP) * dt

INIT Anal_Cancer_TP = 0

INFLOWS:

Develop_Anal_Cancer_TP = (High_Grade_Dysplasia_TP-Dysplasia_Cured)*HG_to_Cancer

Initial_Cancer_TP = Init_Cancer*Anal_HPV_TP

OUTFLOWS:

Anal_Death_Rate_TP = 0.083*Anal_Cancer_TP

Anal_HPV(t) = Anal_HPV(t - dt) + (With_Anal_Infection - Initial_No_Dysplasia - Initial_Low_Grade - Initial_High_Grade - Initial_Cancer - cervical_cancer_deaths) * dt

INIT Anal_HPV = 5555

INFLOWS:

With_Anal_Infection = 5555

OUTFLOWS:

Initial_No_Dysplasia = Init_NO*Anal_HPV

Initial_Low_Grade = Init_LG*Anal_HPV

Initial_High_Grade = Init_HG*Anal_HPV

Initial_Cancer = Init_Cancer*Anal_HPV

cervical_cancer_deaths = Anal_HPV*cervical_death_rate

Anal_HPV_TP(t) = Anal_HPV_TP(t - dt) + (With_Anal_Infection_TP - Initial_No_Dysplasia_TP - Initial_Low_Grade

Ansl_csncer(t) = Ansl_csncer(t - dt) + (Develop_Anal_Cancer + Initial_Cancer - Anal_Death_Rate) * dt

INIT Ansl_csncer = 0

INFLOWS:

Develop_Anal_Cancer = HG_to_Cancer*High_Grade_Dysplasia

Initial_Cancer = Init_Cancer*Anal_HPV

OUTFLOWS:

Anal_Death_Rate = 0.083*Ansl_csncer

died_cervical_cancer(t) = died_cervical_cancer(t - dt) + (cervical_cancer_deaths) * dt

INIT died_cervical_cancer = 0

INFLOWS:

cervical_cancer_deaths = Anal_HPV*cervical_death_rate

died_cervical_cancer_tp(t) = died_cervical_cancer_tp(t - dt) + (cervical_cancer_deaths_5) * dt

INIT died_cervical_cancer_tp = 0

INFLOWS:

cervical_cancer_deaths_5 = Anal_HPV_TP*cervical_cancer_death_rate_2

Dysplasia_Cured(t) = Dysplasia_Cured(t - dt) + (Treatment) * dt

INIT Dysplasia_Cured = 0

INFLOWS:

Treatment = 0.95*High_Grade_Dysplasia_TP

High_Grade_Dysplasia(t) = High_Grade_Dysplasia(t - dt) + (Initial_High_Grade + LG_to_HG_flow + NO_to_HG - Develop_Anal_Cancer - cervical_cancer_deaths_4) * dt

INIT High_Grade_Dysplasia = 0

INFLOWS:

Initial_High_Grade = Init_HG*Anal_HPV

LG_to_HG_flow = Low_Grade_Dysplasia*LG_to_HG -HG_to_LG*High_Grade_Dysplasia

NO_to_HG = No_to_HD*HPV_No_Dysplasa-HD_to_NO*High_Grade_Dysplasia

OUTFLOWS:

Develop_Anal_Cancer = HG_to_Cancer*High_Grade_Dysplasia

cervical_cancer_deaths_4 = High_Grade_Dysplasia*cervical_death_rate

High_Grade_Dysplasia_TP(t) = High_Grade_Dysplasia_TP(t - dt) + (Initial_High_Grade_TP + LG_to_HG_TP + NO_to_HG_TP - Develop_Anal_Cancer_TP - Treatment) * dt

INIT High_Grade_Dysplasia_TP = 0

INFLOWS:

Initial_High_Grade_TP = Init_HG*Anal_HPV_TP

LG_to_HG_TP = Low_Grade_Dysplasia_TP*LG_to_HG -HG_to_LG*High_Grade_Dysplasia_TP

NO_to_HG_TP = HPV_No_Dysplasa_TP*No_to_HD

OUTFLOWS:

Develop_Anal_Cancer_TP = (High_Grade_Dysplasia_TP-Dysplasia_Cured)*HG_to_Cancer

Treatment = 0.95*High_Grade_Dysplasia_TP

HPV_No_Dysplasa(t) = HPV_No_Dysplasa(t - dt) + (Initial_No_Dysplasia - Develops_Low_Grade - NO_to_HG - cervical_cancer_deaths_1) * dt

INIT HPV_No_Dysplasa = 0

INFLOWS:

Initial_No_Dysplasia = Init_NO*Anal_HPV

OUTFLOWS:

Develops_Low_Grade = NO_to_LG*HPV_No_Dysplasa - LG_to_NO*Low_Grade_Dysplasia

NO_to_HG = No_to_HD*HPV_No_Dysplasa-HD_to_NO*High_Grade_Dysplasia

cervical_cancer_deaths_1 = HPV_No_Dysplasa*cervical_death_rate

HPV_No_Dysplasa_TP(t) = HPV_No_Dysplasa_TP(t - dt) + (Initial_No_Dysplasia_TP - Develops_Low_Grade_TP - NO_to_HG_TP - cervical_cancer_deaths_6) * dt

INIT HPV_No_Dysplasa_TP = 0

INFLOWS:

Initial_No_Dysplasia_TP = Init_NO*Anal_HPV_TP

OUTFLOWS:

Develops_Low_Grade_TP = (NO_to_LG*HPV_No_Dysplasa_TP)-LG_to_NO*Low_Grade_Dysplasia_TP

NO_to_HG_TP = HPV_No_Dysplasa_TP*No_to_HD

cervical_cancer_deaths_6 = HPV_No_Dysplasa_TP*cervical_cancer_death_rate_2

Low_Grade_Dysplasia(t) = Low_Grade_Dysplasia(t - dt) + (Develops_Low_Grade + Initial_Low_Grade - LG_to_HG_flow - cervical_cancer_deaths_3) * dt

INIT Low_Grade_Dysplasia = 0

INFLOWS:

Develops_Low_Grade = NO_to_LG*HPV_No_Dysplasa - LG_to_NO*Low_Grade_Dysplasia

Initial_Low_Grade = Init_LG*Anal_HPV

OUTFLOWS:

LG_to_HG_flow = Low_Grade_Dysplasia*LG_to_HG -HG_to_LG*High_Grade_Dysplasia

cervical_cancer_deaths_3 = Low_Grade_Dysplasia*cervical_death_rate

Low_Grade_Dysplasia_TP(t) = Low_Grade_Dysplasia_TP(t - dt) + (Develops_Low_Grade_TP + Initial_Low_Grade_TP - LG_to_HG_TP - cervical_cancer_deeaths_7) * dt

INIT Low_Grade_Dysplasia_TP = 0

INFLOWS:

Develops_Low_Grade_TP = (NO_to_LG*HPV_No_Dysplasa_TP)-LG_to_NO*Low_Grade_Dysplasia_TP

Initial_Low_Grade_TP = Init_LG*Anal_HPV_TP

OUTFLOWS:

LG_to_HG_TP = Low_Grade_Dysplasia_TP*LG_to_HG -HG_to_LG*High_Grade_Dysplasia_TP

cervical_cancer_deeaths_7 = Low_Grade_Dysplasia_TP*cervical_cancer_death_rate_2

UNATTACHED:

cervical_cancer_deaths_8 = High_Grade_Dysplasia_TP*cervical_cancer_death_rate_2

cervical_cancer_death_rate_2 = .04

cervical_death_rate = .04

HD_to_NO = 0.1136

HG_to_Cancer = 0.05

HG_to_LG = 0.22

Init_Cancer = 0.0203

Init_HG = 0.0997

Init_LG = 0.2

Init_NO = 0.68

LG_to_HG = 0.18

LG_to_NO = 0.2265

No_to_HD = 0.04

NO_to_LG = 0.019
